# Patient perspectives on care received at community acupuncture clinics: a qualitative thematic analysis

**DOI:** 10.1186/1472-6882-13-293

**Published:** 2013-10-29

**Authors:** Kimberly M Tippens, Maria T Chao, Erin Connelly, Adrianna Locke

**Affiliations:** 1 Helfgott Research Institute, National College of Natural Medicine, Portland, OR, USA; 2 Osher Center for Integrative Medicine and the Division of General Internal Medicine-San Francisco General Hospital, University of California, San Francisco, USA; 3 Working Class Acupuncture, Portland, OR, USA

**Keywords:** Acupuncture therapy, Complementary therapies, Access to health care, Community acupuncture, Group acupuncture

## Abstract

**Background:**

Community acupuncture is a recent innovation in acupuncture service delivery in the U.S. that aims to improve access to care through low-cost treatments in group-based settings. Patients at community acupuncture clinics represent a broader socioeconomic spectrum and receive more frequent treatments compared to acupuncture users nationwide. As a relatively new model of acupuncture in the U.S., little is known about the experiences of patients at community acupuncture clinics and whether quality of care is compromised through this high-volume model. The aim of this study was to assess patients’ perspectives on the care received through community acupuncture clinics.

**Methods:**

The investigators conducted qualitative, thematic analysis of written comments from an observational, cross-sectional survey of clients of the Working Class Acupuncture clinics in Portland, Oregon. The survey included an open-ended question for respondents to share comments about their experiences with community acupuncture. Comments were received from 265 community acupuncture patients.

**Results:**

Qualitative analysis of written comments identified two primary themes that elucidate patients’ perspectives on quality of care: 1) aspects of health care delivery unique to community acupuncture, and 2) patient engagement in health care. Patients identified unique aspects of community acupuncture, including structures that facilitate access, processes that make treatments more comfortable and effective and holistic outcomes including physical improvements, enhanced quality of life, and empowerment. The group setting, community-based locations, and low cost were highlighted as aspects of this model that allow patients to access acupuncture.

**Conclusions:**

Patients’ perspectives on the values and experiences unique to community acupuncture offer insights on the quality of care received in these settings. The group setting, community-based locations, and low cost of this model potentially reduce access barriers for those who might not otherwise consider using acupuncture. In addition, the community acupuncture model may offer individuals the opportunity for increased frequency of treatments, which raises pertinent questions about the dose–response relationship of acupuncture and health outcomes. This study provides preliminary data for future evaluations of the quality and effectiveness of community acupuncture. Future studies should include the perspectives of patients who initiated, and subsequently, discontinued community acupuncture treatment.

## Background

Over two-thirds of the United States population has used complementary and alternative medicine (CAM) at least once [1]. Acupuncture is a widely recognized system of CAM, used by over 14 million Americans [2]. Despite increasing interest in acupuncture and a growing evidence base for its efficacy in the management of various chronic conditions [3-6], access to providers and the expense of treatment are factors that limit utilization of acupuncture services for many, especially low income and medically underserved populations [7,8]. Acupuncture treatments in the U.S. typically involve 45–60 minute individual consultations and range in cost from $60 to $100 per visit [9]. In 2007, the median out-of-pocket cost per acupuncture visit nationwide was $48, and at least 25% of acupuncture users paid $75 or more per visit [10].

The community acupuncture model aims to directly address economic and other barriers to access through low-cost group-based treatments. Practitioners of this model promote affordable and accessible acupuncture in community settings. Acupuncture is offered in groups for a sliding scale fee of $15-$50. Proof of income is not required or requested. All patients pay cash for their treatments; health insurance is not accepted. Community acupuncture clinics utilize a common space with multiple reclining chairs, which enable practitioners to treat multiple patients at the same time. Patients are encouraged to keep their needles in for as long as they like, leaving the determination of treatment length to the patient. Potential advantages of this group treatment model are increased frequency of visits, a sense of community and empowerment, affordability for the patient, and a sustainable business model for the practitioner [11]. This low-cost, high volume, fee-for-service business model, is designed to allow patients to receive frequent affordable care without reliance on subsidies or practitioner volunteerism. Currently, 192 clinics in North America are members of a multi-stakeholder cooperative of community acupuncture [12]; and an additional 50–100 non-member acupuncture clinics use a similar group-based model [personal communication, Skip VanMeter, POCA Board of Directors].

Recent descriptive studies indicate that, compared to individual acupuncture treatments, the lower costs of the community acupuncture model broaden socioeconomic access [8,13]. The quality of community acupuncture treatments, however, has been questioned because group-based delivery may shorten the length of consultations and limit the acupuncture points and techniques used to treat patients [14]. A growing body of literature suggests that group treatments in conventional health care settings improve access, affordability and quality of care for a number of conditions [15-18]. Additionally, studies from the United Kingdom have assessed the feasibility of group acupuncture treatments in clinical settings, and reported preliminary evidence of effectiveness and acceptability among patients with pain conditions [19-22]. Community acupuncture clinics in the U.S. may accomplish similar outcomes. However, the specific aspects of the community acupuncture model that affect the quality of treatment, from the patient perspective, are unknown. The current study evaluates patient perspectives on the treatment they received in community acupuncture settings.

## Methods

### Study design

A participatory approach was used to establish an academic-community health partnership with the Working Class Acupuncture (WCA) clinic in Portland, Oregon. Academic and community partners collaborated to identify the most relevant research question, design the study, and develop and disseminate the survey instrument. The research team, in collaboration with WCA clinic practitioners, developed a 33-item survey in English, which examined sociodemographic characteristics of WCA clients, conditions treated, reasons for use, and satisfaction with services. Detailed methods and quantitative results, including clients’ socio-demographic factors, health conditions treated at WCA, cost and frequency of treatment were previously reported [8].

The survey included an open-ended question: “Is there anything else you would like to share about your experience receiving acupuncture at WCA?” This question provided respondents with an opportunity to elaborate responses and identify new issues that were not addressed in closed questions. The use of open fields in survey research is common to elicit additional issues of importance when piloting a new instrument, and to offer respondents the opportunity to express their own opinions [23]. All study procedures and instruments were reviewed and approved by the institutional review board of the National College of Natural Medicine.

### Sampling

The study sample consisted of patients of two WCA clinics in Portland, Oregon. Established in 2002, the original WCA clinic is one of the first and largest community acupuncture clinics in the United States. A second WCA clinic site was opened in 2009. Combined, the two WCA clinics provide over 700 acupuncture treatments per week [24]. New and existing patients at both clinics were surveyed for this study.

### Data collection

The paper survey was made available to all clients visiting the community acupuncture clinics for a period of six weeks during December 2009-January 2010. Front desk staff presented the survey to new and returning patients at check-in. Patients were informed that completing the survey was voluntary. The anonymous survey included a consent statement for research in the introductory paragraph. Completed surveys were deposited in a labeled collection box in the clinic waiting area. Of the 500 surveys distributed, 478 completed surveys were returned.

### Data analysis

Written responses to the open-ended survey question were transcribed and analyzed through a series of iterative steps using descriptive coding methods [25,26]. Respondents typically wrote 1–3 sentences in the field provided. We conducted thematic analysis of responses to identify salient themes and topics [27,28]. Thematic analysis was chosen because of its ability to directly represent the descriptions of respondents’ viewpoints, experiences, beliefs, and perceptions [28]. Through these analyses, we used themes as a mode for assigning meaning to topics that were repeated by multiple respondents.

All patient comments were coded individually by three investigators (KMT, MTC, EC) using inductive coding techniques. Rather than assessing inter-rater reliability, the purpose of this approach was to insure that all possible analytic categories were identified and that the team agreed on the final set of codes. Following the initial coding stage, the team developed a revised code set that included new codes, combined repetitive codes and refined some codes to reflect the true sentiment of the associated comments. Using the final agreed-upon coding structure, the research team again independently coded all comments, and then collectively adjudicated discrepancies in the assignment of codes through consensus. Text with multiple concepts described by the respondent could be assigned more than one code. During the latter stage, codes were assigned by each team member to the narrative text using ^©^QSR NVivo 9 qualitative data analysis software (QSR International, Australia). This program facilitates organizing and reviewing coded text for the purposes of qualitative analysis [29].

## Results

Of the 478 completed surveys, 265 included written comments to the open-ended question. We assessed whether respondents who provided qualitative data differed from respondents that did not. The two groups did not differ on any sociodemographic factors, health status, or reasons for seeking care at WCA except for sex (Table 1). The majority of the 265 comments (88%) provided were characterized by the investigators as positive. Those that were not explicitly positive included 22 comments (8%) coded as 'suggestions’, such as patients requesting more information about how acupuncture works or suggestions for clinic staffing and locations; and 10 comments (4%) coded as 'unrelated to acupuncture’, such as feedback about the survey.

**Table 1:** Sociodemographic factors of community acupuncture survey respondents

	**Respondents with qualitative data**	**Respondents without qualitative data**
	**Percent**	**Percent**
	**(n = 265)**	**(n = 213)**
Sex*		
Female	65.1	76.8
**Race/ethnicity**		
Non-Hispanic White	89.2	84.9
African American	1.5	2.3
Latino	4.4	4.6
Asian American	3.4	3.1
Other	1.5	5.0
**Place of birth**		
Foreign born	9.2	6.6
**Education level**		
High school or less	0.5	2.0
Some college	24.0	29.0
College graduate	46.4	39.8
Graduate degree	29.1	29.3
**Household income**		
Less than $35,000	51.8	56.0
$35,000 – $74,999	35.2	33.9
$75,000 or more	13.1	10.1
**Health status**		
Poor or fair	12.4	17.2
Good	39.7	36.0
Very good	36.4	37.2
Excellent	11.5	9.6

*Statistically significant difference between qualitative respondents and non-respondents at p < 0.01.

Analysis of the remaining 233 comments generated 10 central codes clustering under two key themes: (1) aspects of health care delivery unique to community acupuncture and (2) patient engagement in health care. The first theme summarizes comments in which patients discussed their experiences with and perceptions of acupuncture received in a community setting compared to health care received in biomedical settings or other acupuncture settings. The second theme describes patients’ experiences at WCA that have provided them with inspiration, motivation, or a sense of empowerment to take control of their own health care.

### Theme 1: Aspects of health care delivery unique to community acupuncture

Under this theme, patients’ comments highlighted (1) how the community acupuncture model promotes access to health care through cost, scheduling, and setting; and (2) health-related approaches and benefits specific to community acupuncture. Associated codes and comments are presented in Table 2. The ability to afford community acupuncture treatments because of the low cost was identified as an important factor, with cost specifically mentioned in 17% (n=45) of comments. Community acupuncture was described as an accessible option for the uninsured and underinsured, one that was “affordable, approachable and effective.” Patients described the care offered at WCA as an important option for those with little income, no health insurance, and multiple health problems. Several patients specifically commented on the low cost of community acupuncture compared to individual acupuncture treatments, stating that it was the only affordable treatment they could access, or that they could not afford acupuncture otherwise.

The convenience and ease of scheduling were discussed as facilitating factors that improve access to acupuncture services. Patients valued the option to receive treatments when needed, on a walk-in basis. Cost was also mentioned in this context. One patient described community acupuncture treatment as “…so accessible in terms of price and availability of appointments.” Another described scheduling as “easy…because of low cost [and] evening [appointments],” explaining that these factors allow for frequent treatments.

The clinic environment, including the lighting, soft music, and comfortable chairs facilitated patient satisfaction among survey patients. The treatment setting was described as “non-medical,” “warm,” “welcoming,” and “healing.” Patients mentioned feeling relaxed, safe, cared for and respected. In addition, the structure of group treatments and the communal setting were described as therapeutic. As one patient stated, “The 'sense of community’ also changes the way treatment feels…it’s more powerful and comforting.” Another stated, “I love the group sense of well-being, healing and striving for health.”

Patients drew comparisons between acupuncture and conventional biomedicine, describing acupuncture as “safer (without the side effects).” Patients valued prioritization of healing over palliation and the ability to treat multiple conditions simultaneously with acupuncture.

Patients also compared community acupuncture to individual acupuncture treatments. In addition to the low costs and convenience of community acupuncture, many patients commented on the efficient treatment process at the community acupuncture clinic, in which needles are inserted relatively quickly and with concise and focused discussion between the patient and practitioner. The quality or “level of care” received from WCA was described as superior to individual acupuncture treatment received in private settings.

Within this theme, patients attributed decreasing or discontinuing medications for long-standing ailments to the benefits of community acupuncture. A patient stated, “I have been able to stop using all prescribed [medications] for my psoriasis, and keep it under control with acupuncture alone.” Acupuncture was described as a means to alleviate or resolve physical symptoms without the side effects commonly experienced with medications.

**Table 2:** Illustrative comments on aspects of health care delivery unique to community acupuncture

**Codes**	**Quotes**
Appreciating access	*“I think it's great to have this option for people that do not have the funds to pay large fees for this type of medical care, especially for families who need this!!!”*
	*“I love how easy and accessible it is for me AND my family to come - because of low cost, evening appointments. Awesome staff. I come very regularly!”*
Appreciating environment	*“I like the relaxing atmosphere - big chairs and music make it possible to do that.”*
Comparing to conventional medicine	*“I've been seeing doctors for pain for 20 years and not one of them ever indicated a time in the future when I would be 'well’. On my first visit to WCA, my acupuncturist told me how we would progress and that as I got better I would come for fewer treatments. The possibility of getting better was totally mind-blowing.”*
Comparing to other acupuncture	*“… I've been to many acupuncturists, mostly individual practices… At WCA, I trust that the acupuncturists will talk less, give me more space, meet me where I'm at, and charge less!”*
Decreasing medications	*“An answer to prayer for severe chronic pain unaided by other means.* [I have been] *able to reduce the amount of narcotic prescriptions used for pain since I began acupuncture.”*

### Theme 2: Patient engagement in health care

Patients’ experiences with community acupuncture provided them with a source of inspiration, empowerment, and motivation to participate actively in their own health care. Codes and comments associated with this theme are presented in Table 3.

Several comments acknowledged characteristics unique to the WCA mission that support engagement and active participation in one’s care, and reflect philosophical values of personal importance to patients. The business model was described as “revolutionary and progressive,” and a strong political sentiment was reflected in comments such as, “People before profits!” and “American health care system beware!” The mission and philosophy of the clinic appears to encourage and support patient engagement in care by providing a strong sense of allegiance and purpose. As one patient reported, “My love for alternative health approaches brought me here, but the environment, the care and activism kept me coming back.”

Patients placed a high value on practitioners’ skill level and the patient-practitioner relationship. Practitioners were described as, “knowledgeable,” “skilled” and “competent, caring professionals.” One patient stated, “The practitioners feel like family and the treatments have changed my life after it seemed like nothing would.” Practitioners’ skills and ability to perform treatments were not considered separate from their “caring nature,” or their process of offering treatment with encouragement and support. Treatments were described as strongly effective, with words like “helpful” and “powerful” right alongside of “supportive” and “kind.”

Patients reported that care through the community acupuncture clinic promoted feelings of personal control over their care, health and wellness, and they experienced improvements in overall well-being, energy level, and personal perspectives on health. The ability to manage one’s own illness was characterized as an expression of engagement, empowerment, and satisfaction with the care provided. In addition, holistic outcomes such as improved quality of life, mental and emotional healing, and ability to manage stress were commonly reported. One patient described the results of treatment stating, “…I believe it has started me on a path of being conscientious about maintaining overall health.” Others described treatment as “life-changing,” resulting in increased energy levels and overall well-being. As one patient put it, “WCA has added greatly to the over-all 'goodness’ and wellbeing in my life. It helps me do the things I need to do, and want to do.”

**Table 3:** Illustrative comments on patient engagement in health care

**Codes**	**Quotes**
Supporting mission/philosophy	*“I support their economic and political model. I applaud them for putting healthcare and access before economic, academic, and political hierarchies. It takes a lot of integrity and resilience as well as selflessness to practice a style of health care and organization such as theirs in a society that operates from money, greed, competition, and egoism-* [especially] *in such an “elite” field as medicine. The traditional economic model in the U.S. runs on fear and perceptions of security. WCA rises above that and challenges us all- to health!”*
Appreciating practitioners	
*Technical processes:*	*“Your knowledgeable practitioners and affordable fees have cured in three weeks a condition I have been battling for almost a year. Awesome results!”*
*Interpersonal processes:*	*“The level of commitment between practitioner/client is strong. The practitioners’ devotion to helping people is very clear, the community is warm and welcoming and all clients are taking initiative for their well-being.”*
Taking control of health	*“I'm so glad that WCA is here. I feel more empowered as I can make the appointments with relatively short notice, and feel like I have a bit of control over my illnesses.”*
Experiencing multifaceted health benefits	*“I'm just amazed at the remarkable, very real improvement to my physical and emotional well-being. I didn't come in for stress or anything, but have noticed an improvement in my sense of well-being, too.”*
	*“I [cannot] say enough about how much my health has improved w/ treatment at WCA. I have no pain from the neuroma; my TMJ pain is completely gone. My stress from my job has become so manageable with each treatment.”*
Managing own illness	*“It has changed my whole way of thinking about my health and wellness. It gives me a source of hope when experiencing health issues-I know that I can turn to something that will work for pain, stress, allergies, etc.”*

## Discussion

Our study is among the first to describe patient experiences with community acupuncture, a model that has been growing in popularity in the U.S. for the past decade. We identified patient-reported components of access, costs, and quality of community acupuncture. This preliminary investigation has identified a number of areas for future investigation, including further assessment of holistic outcomes of group acupuncture in community settings.

Assessment of the quality of CAM systems of care requires a holistic worldview of the system, encompassing the patient-provider relationship, the treatment provided within that system, and the philosophical context of care [30]. Consistent with this holistic worldview, there are structural and practical aspects of community acupuncture that support patient-centered outcomes. Our findings can be contextualized within Donabedian’s health care quality framework, which describes that high-quality care results when structures, processes, and outcomes of health care are sufficient and functional [31]. This self-selected sample of survey respondents described structural factors of WCA, including affordable payment and ease of scheduling. These structural factors are perceived by patients to improve access to regular and frequent acupuncture treatments, and to increase their control over how and when they access care. Study findings suggest that patients at community acupuncture clinics have a high level of engagement in care. The physical environment and communal setting, along with the enthusiasm garnered by the clinic philosophy and business model, contribute to activation and empowerment among this group of Portland community acupuncture patients. Structures enhancing financial and temporal access serve as facilitating factors of acupuncture use within this model.

In addition, many aspects of the community acupuncture model exemplify a patient-centered approach to care, which is increasingly recognized as a primary dimension of quality and optimal health care delivery [32,33]. Consistent with previous research on patient-centered outcomes associated with CAM [34-36], patients in our study reported benefits such as empowerment, hope, relaxation, mental, emotional and physical health, and improved quality of life. Patients expressed value for practitioners, suggesting that the therapeutic relationship is not compromised despite the fact that verbal communication is kept to a minimum in the community acupuncture model.

The generalizability of these analyses is limited by the convenience sample employed for the study. A known limitation of qualitative research is that findings may be unique to the persons included in the research study, and may not generalize to other populations [37]. The sampling method that we used did not allow for accessing patients who initiated, but did not continue receiving acupuncture at WCA clinics. As a result, our sample is more likely to represent individuals who are satisfied with the care they received at community acupuncture clinics. Additionally, data analyzed in this paper were limited to responses to a single open-ended survey item. Nevertheless, this collection of comments from a sample of satisfied community acupuncture patients provides insight into the unique dimensions of care at Portland community acupuncture clinics, reasons for choosing to receive care within this acupuncture delivery model, and patient perspectives on quality of care. While there are limitations to using open-ended questions on surveys, including the lack of specificity in responses and analytic challenges, these do not outweigh the benefits of inviting individuals’ unique perspectives and experiences in their own words [23]. Future studies using our instrument should include explicit plans to optimize the value of data provided in written responses.

The generalizability of our findings may also be limited by the study setting, where the use of acupuncture and other CAM are relatively accepted in the sociocultural context of Portland. Nonetheless, our analyses identified structural and practical aspects of the community acupuncture model – including low cost treatments, ease of scheduling, and community-based locations – that may be useful to improve access to acupuncture in areas where it is less prevalent.

Importantly, patients’ perceptions that quality is not compromised through community acupuncture are relevant to the discussion of CAM models that may address gaps in health care. In the United States, CAM utilization is associated with poor health status and with having unmet health care needs or delayed care due to cost [38,39]. One out of four WCA survey respondents was uninsured; a factor known to be associated with having unmet health care needs [40]. The community acupuncture model, while not a full substitute for basic healthcare, is potentially filling an important gap for uninsured, underinsured, and medically underserved populations in Portland.

## Conclusions

In this study, Portland community acupuncture patients perceive their care as high quality. Themes that emerged from these analyses highlight how the community acupuncture model facilitates access to acupuncture. Patients mentioned the facilities and material resources that support the provision of care, such as aspects of the physical setting and relaxing atmosphere. Patients identified both technical and interpersonal processes of care received at WCA that support patient engagement. They described their processes for accessing care as empowering, allowing them to exercise control over when and how they are treated. Our findings suggest that the group setting, community-based locations, and low cost of this model do not compromise quality of care, and potentially reduce access barriers for those who might not otherwise consider acupuncture. In addition, the community acupuncture model may offer individuals the opportunity for increased frequency of treatments, which raises pertinent questions about the dose–response relationship of acupuncture and health outcomes. Further work is needed to examine aspects of access, costs, and quality of care with larger, more representative samples of community acupuncture patients from multiple clinics. Future studies should also include the perspectives and experiences of patients who initiated, and subsequently, terminated treatment through community acupuncture clinics due to dissatisfaction.

## Competing interests

AL participated in this study as a student research assistant while studying Chinese medicine at the National College of Natural Medicine. Since completion of the study, AL received a Master’s degree in acupuncture and obtained employment as an acupuncturist for Working Class Acupuncture in Portland, Oregon. None of the other authors has competing financial interests to report.

## Authors’ contributions

KMT contributed to study conception and design, implementation, analysis and interpretation, and led the manuscript preparation. MTC contributed to study conception and design, analysis and interpretation, and drafts of the manuscripts. KMT, MTC, and EC conducted all coding of the data. EC and AL contributed to study implementation, analyses and interpretation, and manuscript preparation. All authors read and approved the final manuscript.

## Pre-publication history

The pre-publication history for this paper can be accessed here:

http://www.biomedcentral.com/1472-6882/13/293/prepub
